# Phenotype-Specific Mitochondrial Responses to Mediterranean Diet and Exercise in Elderly Obesity

**DOI:** 10.3390/nu18030475

**Published:** 2026-02-01

**Authors:** Paloma Carrillo-Fernández, María Ángeles Silva-Soto, Rocío Gallego-Durán, Elena Medina-Jimenez, Alberto Vilches-Pérez, Juan Francisco Mogaburo-Alba, Tania E. Saez-Lancellotti, Ana Navarro-Sanz, Nuria Prieto-Lain, Ana Isabel Gómez-Hernández, Sergio Jansen-Chaparro, Douglas Maya-Miles, Manuel Romero-Gomez, Ricardo Gómez-Huelgas, María Rosa Bernal-Lopez

**Affiliations:** 1 UCM Digestive Diseases, Virgen del Rocío University Hospital, Instituto de Biomedicina de Sevilla (HUVR/CSIC/US), Department of Medicine, University of Seville, 41004 Seville, Spain; pcarrillo-ibis@us.es (P.C.-F.); rgallego-ibis@us.es (R.G.-D.); dmaya-ibis@us.es (D.M.-M.); mromerogomez@us.es (M.R.-G.); 2 Department of Internal Medicine, Regional University Hospital of Málaga, Instituto de Investigación Biomédica de Málaga (IBIMA Plataforma BIONAND), University of Málaga, 29009 Málaga, Spain; angeles.silva@ibima.eu (M.Á.S.-S.); elena.mj2000@gmail.com (E.M.-J.); eslancellotti@gmail.com (T.E.S.-L.); prietolain@hotmail.com (N.P.-L.); anabelgh96@hotmail.com (A.I.G.-H.); sjansenc@gmail.com (S.J.-C.); ricardogomezhuelgas@hotmail.com (R.G.-H.); 3 Centro de Investigación Biomédica en Red en Enfermedades Hepáticas y Digestivas (CIBEREHD), Instituto de Salud Carlos III, 28029 Madrid, Spain; 4 Endocrinology and Nutrition Department, Virgen de la Victoria University Hospital, Instituto de Investigación Biomédica de Málaga (IBIMA Plataforma BIONAND), 29010 Málaga, Spain; alberto_v4@hotmail.com; 5 Sports Area, Sport Medicine, Málaga City Hall, 29006 Málaga, Spain; ansanz@malaga.eu; 6 Centro de Investigación Biomédica en Red en Fisiopatología de la Obesidad y la Nutrición (CIBEROBN), Instituto de Salud Carlos III, 28029 Madrid, Spain

**Keywords:** obesity, elderly, lifestyle, mitochondria, exercise, mediterranean diet

## Abstract

**Background/Objectives:** While excessive body fat is commonly linked to metabolic disorders (metabolically unhealthy obesity, MUO), a subset of individuals remain metabolic healthy despite obesity (metabolically healthy obesity, MHO). This work aims to determine how these phenotypes influence responses to lifestyle modification (LSM) in older adults. **Methods:** A 12-month lifestyle modification (LSM) intervention based on the Mediterranean Diet (MedDiet) and regular physical activity (PA) was conducted in 43 older adults (70% women) classified according to World Health Organization (WHO) criteria as MHO (22 subjects) or MUO (21 subjects). Clinical, dietary, and PA parameters were assessed at baseline and follow-up. Peripheral blood mononuclear cells were analyzed for mitochondrial fusion (OPA1, MFN2), mitophagy (PINK1), biogenesis (TFAM), and the respiratory chain (COX IV) using Western blot and RT-qPCR techniques. **Results:** At baseline, MUO showed significant lower OPA1-L, MFN2, and TFAM along with MFN2 degradation products and PINK1 accumulation. After 12 months of LSM, MUO participants exhibited greater metabolic profile improvements, such as significantly reduced MFN2 degradation products and higher COX IV. Changes in mitochondrial proteins were associated with nutrient intake and PA and clinical parameters with phenotype-specific patterns. In MUO, protein and cholesterol intake improved MFN2 fusion (rho = 0.446, *p* = 0.043; rho = 0.581, *p* = 0.006), while carbohydrates were negatively associated with OPA1 in MHO (rho = −0.596, *p* = 0.025). PA was positively related to fusion proteins in both phenotypes. Clinically, significant improvements in BMI, waist circumference, and HDL were found in MUO but not in MHO. **Conclusions:** Older adults with obesity show phenotype-specific mitochondrial impairments that shape distinct responses to LSM, highlighting the relevance of tailoring LSM interventions by metabolic phenotype.

## 1. Introduction

Overweight and obesity prevalences are rising globally and now affect about 60% of all adults in Europe [1]. According to the latest World Obesity Atlas, over four billion people may be overweight by 2035 [2]. This multifaceted condition impairs individuals’ health and places a significant burden on healthcare systems worldwide.

Excessive adiposity is closely associated with the onset of metabolic disorders such as type 2 diabetes mellitus (T2DM), dyslipidemia, hypertension, chronic inflammation, and insulin resistance, also known as a metabolically unhealthy obesity (MUO) profile. On the other hand, some individuals with obesity are more resistant to the development of these abnormalities and do not present with any of them. This is known as a metabolically healthy obesity (MHO) profile. These individuals even have a lower risk of cardiovascular disease [3]. One of the main obstacles in defining the MHO and MUO phenotypes is the wide heterogeneity of metabolic criteria that can be considered. Some studies use insulin sensitivity, while others use blood pressure or lipid profile. This poses a challenge in metabolic research [4]. This study uses criteria that take into account four metabolic abnormalities (blood pressure, triglyceride levels, high-density lipoprotein (HDL) cholesterol levels, and fasting plasma glucose). These parameters have been widely used in other studies and with a variety of populations [5].

Lifestyle modification (LSM)—including dietary improvement and increased physical activity (PA)—is the cornerstone of obesity care and is key in reducing its metabolic consequences. Current guidelines recommend healthier dietary choices, mainly through caloric restriction, together with an increase in PA and stress management to mitigate the risk of obesity and promote weight management [6]. In particular, the combination of the Mediterranean diet (MedDiet) and regular PA have shown beneficial effects on weight, inflammation, and insulin sensitivity in several populations [7].

Mitochondrial dynamics have been considered as a potential therapeutic target in a wide range of human diseases, including obesity and aging [8]. Several studies have previously linked metabolic disorders to mitochondrial dysfunction. Obesity and T2DM have been associated with defects in some proteins involved in mitochondrial dynamics, such as mitofusin 2 (MFN2) and optic atrophy type 1 (Opa1); both proteins participate in mitochondrial fusion [9]. Furthermore, other proteins play an important role in regulating mitochondrial biogenesis, such as PTEN-induced kinase 1 (PINK1), involved in mitophagy, or mitochondrial transcription factor A (TFAM), implicated in mitochondrial DNA (mtDNA) replication and transcription [10,11]. Finally, mitochondrial activity is known to be reduced by 40% in the older adult population when compared to the younger population, so aging may be considered an additional risk factor for mitochondrial dysfunction [12].

Considering this, the main aim of this observational cohort study was to evaluate the effect of a 12-month LSM based on the MedDiet and PA on mitochondrial dynamics and function in peripheral blood mononuclear cells (PBMCs) and the clinical outcomes of older adults with obesity. In addition, the responses of individuals with MHO and MUO phenotypes were compared to determine whether mitochondrial remodeling differs according to metabolic health status.

## 2. Materials and Methods

### 2.1. Study Design

This study is a secondary analysis of a subset of participants previously included in an interventional trial [13]. Forty-three elderly individuals were selected for mitochondrial analyses according to the availability of PBMCs and completion of follow-up. Inclusion criteria were (i) men and women aged ≥ 65 years and (ii) obesity (BMI ≥ 30–<40 kg/m^2^).

According to WHO criteria, they were divided into MHO (n = 22) and MUO (n = 21) populations. The metabolically healthy obesity (MHO) phenotype was defined as the presence of ≤1 of the following 4 cardiometabolic abnormalities:

(1) Systolic blood pressure ≥ 130 and/or diastolic blood pressure ≥ 85 mmHg (or antihypertensive drug treatment in a patient with a history of hypertension);
(2) Triglycerides ≥ 150 mg/dL (or treatment with fibrates, nicotinic acid, or ω-3 fatty acids);
(3) High-density lipoprotein (HDL) cholesterol < 40 mg/dL in men or <50 mg/dL in women (or treatment with fibrates or nicotinic acid);
(4) Fasting plasma glucose ≥ 100 mg/dL (or previous diagnosis of diabetes or treatment with antidiabetic drugs).

The metabolically unhealthy obesity (MUO) phenotype was defined as the presence of ≥2 of the aforementioned cardiometabolic abnormalities.

### 2.2. Ethical Considerations

This study was conducted in accordance with the Declaration of Helsinki. Its design and methods were approved by the Andalusian Research Ethics Committee (ref: PI18/00766-260718 and PI21/00465-161221), part of the Ministry of Health and Consumer Affairs of the Andalusian Regional Government (Spain). Written informed consent was obtained from all participants prior to inclusion in the study.

### 2.3. Lifestyle Intervention and Clinical and Anthropometric Assessment

Participants were evaluated at baseline and following a 12-month intervention period. Every three months, trained clinical staff performed anthropometric assessments that included weight, height, BMI, waist circumference, and blood pressure measurements as well as the collection of blood samples. To assess the impact of the lifestyle intervention, standardized questionnaires were administered at each visit to monitor dietary intake, encourage the MedDiet, and promote regular PA. Participants completed five scheduled clinical visits (baseline, 3, 6, 9, and 12 months), which were conducted by trained dietitians who followed standardized procedures. The dietary intervention emphasized adherence to the MedDiet and food quality without specific caloric restriction. To facilitate compliance, participants received standardized written materials, including weekly menus and MedDiet-based recipes. Regarding physical activity, participants were encouraged to perform at least 150 min of moderate PA per week [13]. MedDiet adherence was assessed using the 14-item Mediterranean Diet Adherence Screener (MEDAS), which classifies adherence as low (0–5), moderate (6–9), or high (≥10) [14]. PA levels were assessed using the Rapid Assessment of Physical Activity (RAPA) questionnaire as sedentary (1), underactive (2), regular underactive (light activities) (3), regular underactive (4–5), or regular active (6–7) [15].

### 2.4. Isolation of PBMCs

PBMCs were obtained from whole blood in EDTA tubes after centrifugation to separate plasma and serum. They were isolated from the buffy coat of the anticoagulant-treated blood using Ficoll-standard density gradient centrifugation (1800 rpm; 25 min). Then, samples were washed twice with PBS and centrifuged (1800 rpm, 10 min) and the final fraction of PBMCs was stored in FBS with DMSO 20% at −80 °C. The entire heterogeneous pool of PBMCs was used for analysis.

### 2.5. RNA and Protein Extraction

RNA and proteins from PBMCs were obtained by the TRIzol-based extraction method. Briefly, TRIzol was added to samples and incubated for 10 min at room temperature, followed by incubation in chloroform for 15 min at room temperature. Then, the colorless aqueous phase containing RNA was transferred to sterile tubes and the phase containing DNA and proteins was preserved. For RNA extraction, samples were incubated with isopropyl alcohol for 10 min at room temperature and washed with EtOH 75%. The pellet containing RNA was allowed to dry and then dissolved in RNase-Free Water and incubated at 55–60 °C for 10 min. Finally, RNA was stored at −80 °C until use. For protein extraction, samples were centrifuged, and the pellet was allowed to dry and then dissolved in SDS 1% (55–60 °C for 10 min). The samples were sonicated and the amount of protein was quantified with a Pierce™ BCA Protein Assay Kit (Ref.23225, Merck, Darmstadt, Germany) and cryopreserved until use.

### 2.6. Western Blot for Mitochondria-Related Proteins Analysis

The expression of MFN2, Opa1, PINK1, TFAM, and COX IV was measured in PBMCs via Western blot at baseline and after the 12-month LSM, using beta actin as the housekeeping gene. Protein samples were subjected to SDS-PAGE electrophoresis using a Mini-PROTEAN Tetra Vertical Electrophoresis Cell (Bio-Rad, Hercules, CA, USA). Proteins were transferred to PVDF membranes in an Invitrogen™ iBlot™ 2 Gel Transfer Device. After blocking in TBS-T containing 5% nonfat dry milk, membranes were hybridized with primary antibodies overnight at 4 °C. The primary and secondary antibodies used in this work are summarized in Table S1. Following one hour of incubation with horseradish peroxidase-conjugated secondary antibodies at room temperature, immunoreactive bands were detected with the Immobilon ECL western Millipore (p90720, Darmstadt, Germany) in an ImageQuant LAS 4000 mini Biomolecular Imager (GE Healthcare, Chicago, IL, USA) and quantified by Fiji software (version 1.1). Protein Precision Plus (Bio-Rad) was used as the standard and data were expressed as protein-to-actin ratios.

### 2.7. Quantitative Real-Time PCR (RT-qPCR)

RNA from samples were measured in a NanoDrop 2000 spectrophotometer (Thermo-Fisher Scientific, Waltham, MA, USA). In total, 30 ng of total RNA per sample was used and RT and qPCR reactions were carried out in duplicate using the KAPA SYBR^®^ FAST One-Step (SF1UKB, Roche, Basel, Switzerland) in an Applied Biosystems 7500 Real-Time PCR System. Data were normalized to GAPDH expression. Primers for qPCR analysis (KiCqStart™ SYBR Green Primers Predesigned) were obtained from Sigma-Aldrich (St. Louis, MO, USA) and are listed in Table S2.

### 2.8. Statistical Analysis

SPSS Statistics 25 and GraphPad Prism 9.0 were used for the statistical analysis. Normality was assessed using the Shapiro–Wilk test. Paired Student’s *t*-tests or Wilcoxon signed-rank tests were applied. Comparisons between groups (MHO vs. MUO) were assessed using unpaired *t*-tests or Mann–Whitney U tests. Only participants with paired baseline and follow-up data were included (complete-case analysis). Sample size may vary across outcomes due to missing clinical or molecular data. Data are expressed as mean ± SEM. Cohen’s d was calculated to estimate effect sizes (small ≥ 0.2, moderate ≥ 0.5, and large ≥ 0.8). Correlations between changes (Δ) in clinical, nutritional, PA, and mitochondrial variables were assessed using Spearman’s rho. Significance was defined as *p* < 0.05 (two-tailed).

## 3. Results

### 3.1. Anthropometric and Clinical Changes After a 12-Month LSM Program

At baseline, the cohort age (N = 43) was 70 ± 4 years, with an average body weight of 84.6 ± 11.2 kg and a BMI of 33.7 ± 3.2 kg/m^2^. Females represented 69.8% of the sample. The MHO (N = 22) and MUO (N = 21) groups showed comparable proportions in age, weight, BMI, and number of females (~70%) (Table 1). After the 12-month LSM, the MHO had maintained stable anthropometric values while the MUO saw significant decreases in weight (−2.79 kg, Cohen’s d = −0.88, *p* < 0.001), BMI (−0.82 kg/m^2^, Cohen’s d = −0.70, *p* = 0.005), and waist circumference (−4.38 cm, Cohen’s d = −0.80, *p* = 0.001), indicating a large and meaningful effect in this population. HDL values increased significantly in both MHO (+11.77 mg/dL, Cohen’s d = 0.40, Wilcoxon *p* = 0.003) and MUO participants (+2.14 mg/dL, Cohen’s d = 0.56, Wilcoxon *p* = 0.018). The rest of the analytical parameters remained unchanged.

Adherence to the MedDiet and PA levels at baseline and after 12 months of LSM are summarized in Table 2. MedDiet adherence significantly increased after LSM in both phenotypes, with a greater magnitude of change observed in MUO participants. This improvement was reflected both in higher mean MEDAS scores and in a shift from low and moderate adherence categories toward high adherence.

In contrast, changes in PA assessed by the RAPA questionnaire differed by phenotype. While MUO participants showed a modest increase in the RAPA score and a reduction in sedentary behavior, MHO participants exhibited a decrease in mean RAPA score over the intervention period. Overall, PA levels displayed substantial inter-individual variability, as reflected by the distribution of RAPA categories before and after lifestyle modification.

### 3.2. Mitochondrial Dynamics Are Impaired in MUO at Baseline

**Figure 1:**
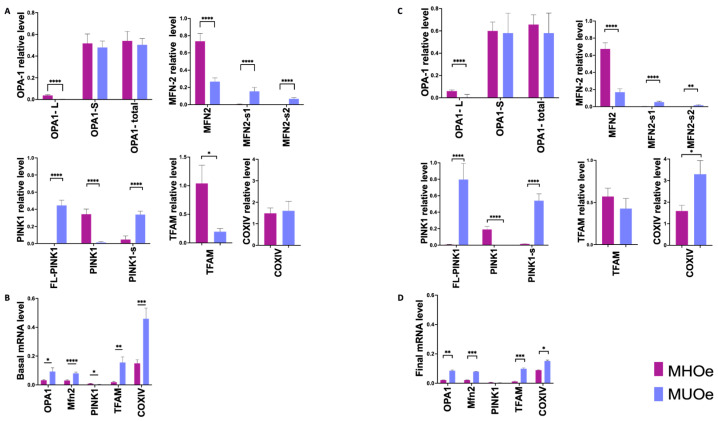
Mitochondrial-related protein differences between MHO and MUO PBMCs determined by Western blot and RT-qPCR. Panels (**A**,**B**) depict baseline cross-sectional comparisons between metabolic phenotypes. Panels (**C**,**D**) show comparisons between phenotypes after 12 months of lifestyle modification. Data are expressed as mean ± SEM. (* *p* < 0.05, ** *p* < 0.01, *** *p* < 0.001, **** *p* < 0.0001).

Baseline analyses revealed marked phenotype-specific differences in mitochondrial dynamics prior to the LSM. MUO participants displayed markedly lower OPA1 and MFN2 protein levels. Specifically, OPA1 long form (OPA1-L, 100 kDa) was ten times lower (*p* < 0.0001) and OPA1 short form (OPA1-S, 80 kDa) 1.2 times lower (non-significant). MFN2 was 2.7 times lower in MUO (*p* < 0.0001) (Figure 1A). Interestingly, two smaller MFN2 bands—about 60 and 45 kDa (MFN2-s1 and -s2, respectively)—were found exclusively in MUO. Despite the fact that OPA1 and MFN2 protein levels were notably lower in MUO, their mRNA levels were statistically higher compared to MHO (Figure 1B).

Regarding mitophagy-related markers, no PINK1 full-length band (FL-PINK1, 65 kDa) was detected in MHO samples (*p* < 0.0001), whereas an increase in mature PINK1 band (55 kDa) and an additional processed band of about 45 kDa (PINK1-s) was observed in this group. In contrast, MUO exhibited two pronounced bands corresponding to FL-PINK1 and the processed PINK1-s. Notably, PINK1 mRNA expression was significantly lower in MUO compared to MHO (*p* = 0.012), despite the accumulation of its protein forms.

Mitochondrial biogenesis was also diminished in MUO, with a 3.2-fold reduction in TFAM when compared to MHO (*p* = 0.001). However, they expressed higher levels of TFAM mRNA when compared to MHO (*p* < 0.001). No significant differences in the COX IV protein were observed at baseline. However, MUO expressed significantly higher levels of COX IV mRNA than MHO (*p* < 0.001).

After the LSM, most differences in protein levels between MHO and MUO were maintained, except for TFAM, which decreased in MHO, and COX IV, which increased in MUO (Figure 1C). Regarding mRNA levels, although there was a general decrease in MUO participants compared to baseline, it was not enough to reach the lower MHO values (Figure 1D).

Notably, several mitochondrial markers showed a clear dissociation between mRNA and protein expression. Despite reduced protein levels of OPA1, MFN2, TFAM, and COX IV in participants with MUO, their mRNA expression was often increased, suggesting the presence of compensatory transcriptional responses and post-transcriptional regulatory mechanisms.

### 3.3. LSM Enhances the Mitochondrial Fusion Profile by Increasing OPA1-L in Both Metabolic Phenotypes After 12 Months of Intervention

**Figure 2:**
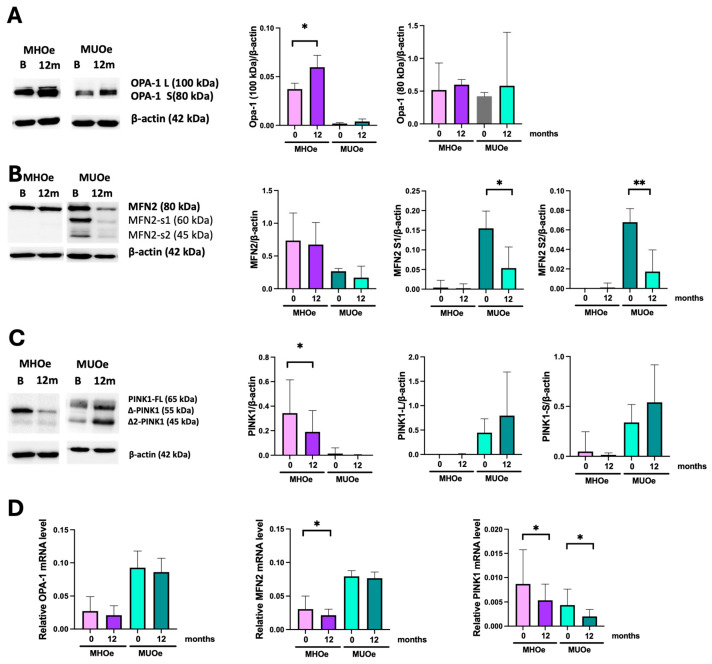
Representative Western blot and relative quantification of (**A**) OPA1, (**B**) MFN2, and (**C**) PINK1, (**D**) RT-qPCR analysis of OPA1, MFN2, and PINK1 mRNA relative expression in MHO and MUO PBMCs at baseline and after a 12-month LSM intervention (* *p* < 0.05, ** *p* < 0.01). Data are expressed as mean ± SEM.

Longitudinal analyses were then performed to assess changes in mitochondrial dynamics and function following the 12-month LSM. Two OPA1 bands of 100 kDa (OPA1-L) and 80 kDa (OPA1-S) were found in both populations, although the larger form was poorly detected in MUO. After the LSM, OPA1-L, increased in both MHO (1.6-fold vs. baseline, *p* = 0.04) and MUO (2-fold vs. baseline, *p* = 0.28) (Figure 2A), and OPA1-S increased modestly (1.16-fold, *p* = n.s.) in MHO and remained stable in MUO. OPA1 mRNA expression was found to be decreased in both groups after the intervention but did not reach statistical significance (Figure 2D).

### 3.4. LSM Enhances the Mitochondrial Fusion Profile in MUO by Decreasing MFN2 Degradation

Regarding MFN2, a different pattern of bands was observed between the groups. In MHO, only the canonical 80 kDa band was detected, with a non-significant decrease after the intervention. MUO showed a banded pattern with two additional bands of about 60 and 45 kDa (Figure 2B), which are considered putative degradation products of MFN2. These bands were significantly lower after the LSM (*p* = 0.02 and *p* = 0.004, respectively). The 80 kDa band in MUO showed a non-significant trend toward reduction (*p* = 0.06). No significant changes were observed in MFN2 mRNA expression in either group following the intervention (Figure 2D).

### 3.5. LSM Modulates Mitophagy Through PINK1 Regulation in MHO but Not in MUO

In MHO, the cytosolic processed mature PINK1 (55 kDa) was significantly decreased after the intervention (*p* = 0.002), while the FL-PINK1 precursor (65 kDa) remained undetectable (Figure 2C). Additionally, they presented another processed form (PINK1-s, 45 kDa) that did not show changes after the LSM. In line with these results, PINK1 mRNA showed a post-interventional decrease in this group (*p* = 0.035) (Figure 2D). In contrast, PINK-1 was weakly detected in MUO, which showed more prominent bands of FL-PINK1 and PINK1-s, which did not see significant changes after the LSM.

### 3.6. TFAM Expression Remains Unchanged After LSM

No differences were observed between baseline and after the 12-month LSM program in TFAM protein expression or mRNA levels (Figure S1).

### 3.7. LSM Increased the Mitochondrial Respiratory Chain Activity in MUO

**Figure 3:**
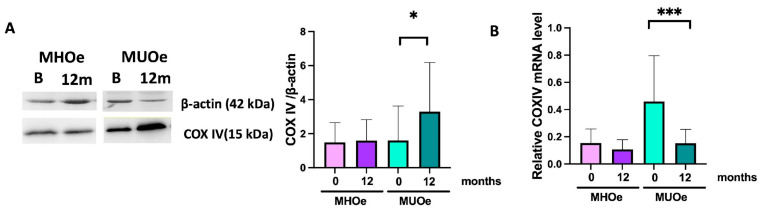
(**A**) Representative Western blot and relative quantification of COX IV and (**B**) RT-qPCR analysis of COX IV mRNA relative expression in MHO and MUHO PBMCs at baseline and after a 12-month LSM intervention (*, *p* <0.05; ***, *p* < 0.001). Data are expressed as mean ± SEM.

After the LSM, MUO showed a significant increase in the COX IV protein (Figure 3A), accompanied by a significant decrease in COX IV mRNA expression (Figure 3B). No changes were found in either COX IV protein or mRNA levels in MHO.

### 3.8. Changes in Mitochondrial-Related Proteins Are Associated with Nutrient Intake and PA

**Figure 4:**
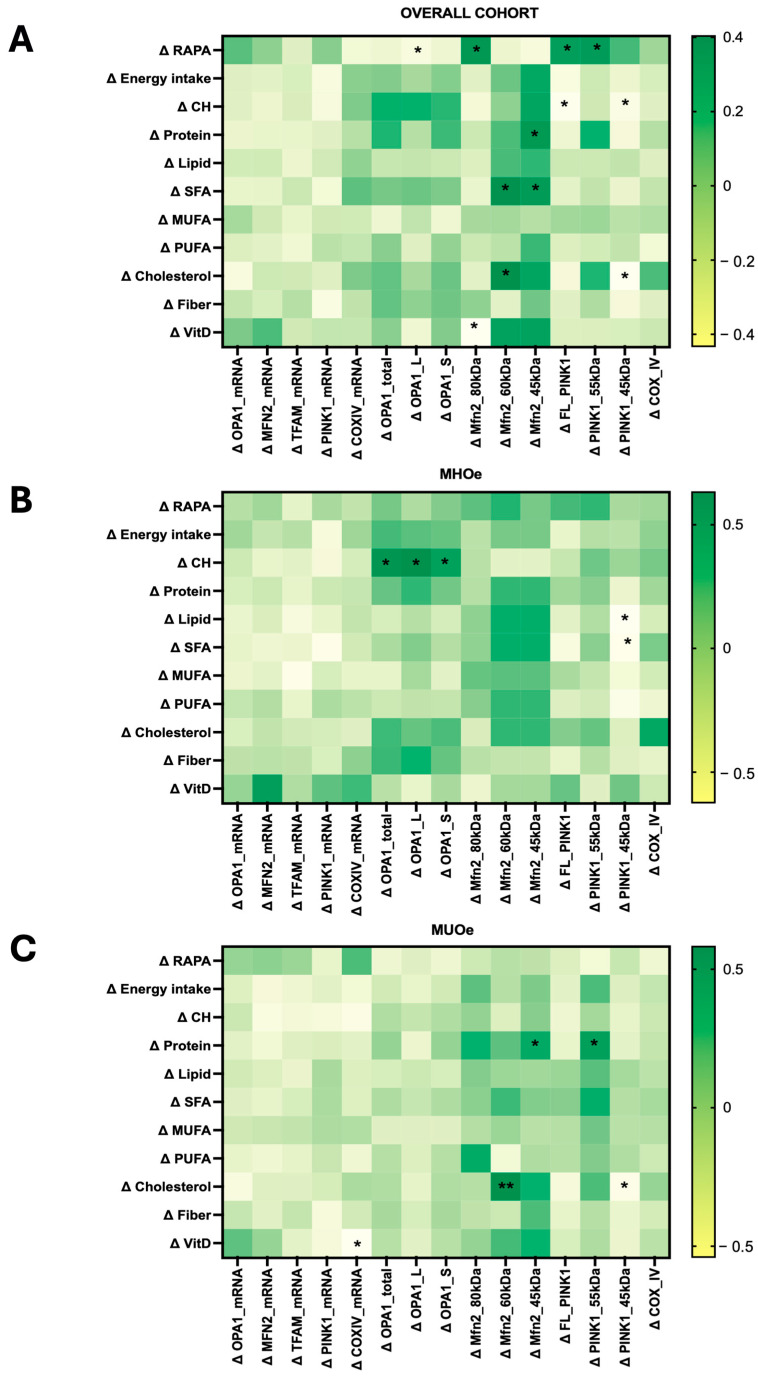
Heatmap showing Spearman correlations between changes in mitochondrial marker levels and PA and nutrient intake in (**A**) overall cohort, (**B**) MHO, and (**C**) MUO. Correlation analyses in subgroup panels are exploratory due to limited sample size and should be interpreted with caution. Correlation coefficients (rho) with statistically significant correlations indicated by asterisks (* *p* < 0.05, ** *p* < 0.01). Abbreviations: CH: Carbohydrates; MHO: Metabolically Healthy Obese older adults; MUFA: Monounsaturated Fatty Acids; MUO: Metabolically Unhealthy Obese older adults; PUFA: Polyunsaturated Fatty Acids; RAPA: Rapid Assessment of Physical Activity; SFA: Saturated Fatty Acids; Vit D: Vitamin D.

Overall nutrient intake was found to correlate with both fusion MFN2 and mitophagy PINK1 markers (Figure 4). In the overall cohort, changes in vitamin D intake inversely correlated with the 80 kDa band of MFN2 (rho = −0.433, N = 35, 95% CI [−0.6 to 0.06]; *p* = 0.009). The smallest form of MFN2 (45 kDa) correlated with protein (rho = 0.368, N = 35, 95% CI [−0.01 to 0.67]; *p* = 0.030) and SFA (rho = 0.362, N = 35, 95% CI [0.02 to 0.63]; *p* = 0.033) intake. Higher changes in carbohydrate intake were associated with reduced levels of FL-PINK1 (rho = −0.409, N = 35, 95% CI [−0.68 to −0.05]; *p* = 0.015) and mature PINK1 (rho = −0.352, N = 35, 95% CI [−0.63 to −0.02]; *p* = 0.038). Likewise, increased cholesterol intake negatively correlated to PINK1-s (rho = −0.429, N = 35, 95% CI [−0.67 to −0.12]; *p* = 0.010).

In MHO, carbohydrate intake was positively associated with total OPA1 protein (rho = 0.596, N = 14, 95% CI [0.06 to 0.9]; *p* = 0.025), whereas total and saturated fat intake correlated inversely with mature PINK1 (rho = −0.624, N = 14, 95% CI [−0.92 to −0.11]; *p* = 0.017; rho = −0.569, N = 14, 95% CI [−0.90 to −0.05]; *p* = 0.034). Vitamin D intake tended to correlate with MFN2 mRNA (rho = 0.549, N = 13, 95% CI [−0.00 to 0.87]; *p* = 0.052).

In MUO, vitamin D intake was inversely associated with COX IV protein (rho = −0.540, N = 21, 95% CI [−0.85 to −0.10]; *p* = 0.014), while cholesterol intake correlated positively with MFN2-s1 (rho = 0.581, 95% CI [−0.24 to 0.72]; *p* = 0.006) and negatively with PINK1-s (rho = −0.461, N = 21, 95% CI [−0.73 to −0.08]; *p* = 0.035). Protein intake showed positive associations with MFN2-s2 (rho = 0.446, N = 21, 95% CI [−0.05 to 0.84]; *p* = 0.043) and mature PINK1 (rho = 0.504, N = 21, 95% CI [0.07 to 0.77]; *p* = 0.020).

Increased PA (ΔRAPA) correlated with ΔOPA1-L (rho = −0.31, N = 43, 95% CI [−0.57 to 0.00]; *p* = 0.039), ΔMFN2 (80 kDa) (rho = 0.37, N = 43, 95% CI [0.10 to 0.68]; *p* = 0.014), ΔFL-PINK1 (rho = 0.35, N = 43, 95% CI [0.07 to 0.60]; *p* = 0.021), and Δ mature PINK1 (rho = 0.359, N = 43, 95% CI [0.01 to 0.62]; *p* = 0.018) proteins.

### 3.9. Changes in Mitochondrial-Related Proteins After LSM Correlate with Several Metabolic and Clinical Outcomes

**Figure 5:**
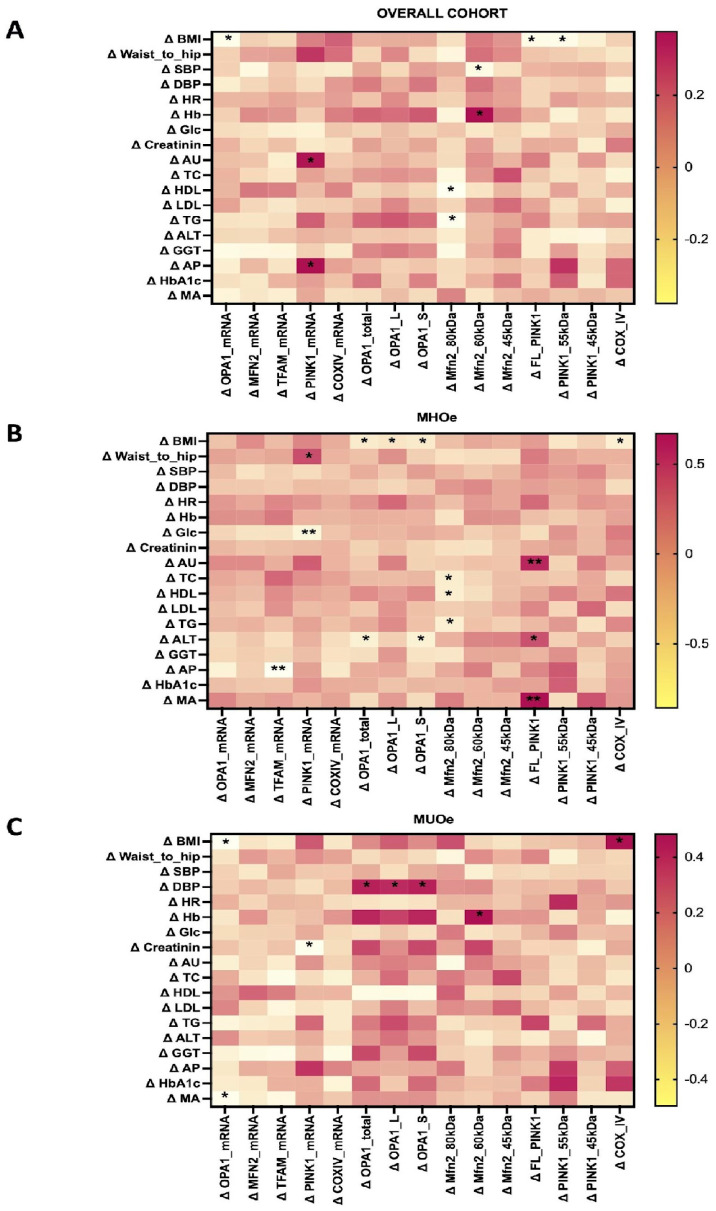
Heatmap showing Spearman correlations between changes in mitochondrial markers levels and clinical outcomes in (**A**) overall cohort, (**B**) MHO, and (**C**) MUO. Correlation analyses in subgroup panels are exploratory due to limited sample size and should be interpreted with caution. Correlation coefficients (rho) with statistically significant correlations indicated by asterisks (* *p* < 0.05, ** *p* < 0.01). Abbreviations: ALT: Alanine Transaminase; AP: Alkaline Phosphatase; UA: Uric Acid; BMI: Body Mass Index; DBP: Diastolic Blood Pressure; GGT: Gamma-Glutamyl Transaminase; Glc: Glucose; Hb: Hemoglobin; HbAc1: glycated hemoglobin; HDL: high-Density Lipoprotein; HR: Heart rate; LDL: Low-Density Lipoprotein; MA: Microalbuminuria; SBP: Systolic Blood Pressure; TC: Total Cholesterol; TG: Triglycerides; UA: Uric Acid.

Mitochondrial changes correlated with multiple metabolic parameters (Figure 5). Variations in OPA1 mRNA correlated negatively with BMI decreases in the total cohort (rho = −0.340, N = 41, 95% CI [−0.64 to −0.60]; *p* = 0.030) and MUO (rho = −0.496, N = 20, 95% CI [−0.80 to −0.01]; *p* = 0.026) groups, and with Δ microalbuminuria in MUO (rho = −0.468, N = 20, 95% CI [−0.80 to 0.00]; *p* = 0.038). In MHO, Δ OPA1-S correlated negatively with ΔBMI (rho = −0.426, N = 22, 95% CI [−0.70 to −0.01]; *p* = 0.048) and with Δ ALT (rho = −0.531, N = 20, 95% CI [−0.81 to −0.11]; *p* = 0.016). In contrast, all forms of OPA1 protein correlated positively with Δ DBP in MUO (rho = 0.439, N = 21, 95% CI [0.02 to 0.73]; *p* = 0.047).

Moreover, Δ MFN2 (80 kDa) showed consistent negative correlations with lipid parameters in the total and MHO cohorts; Δ HDL (rho = −0.378, N = 41, 95% CI [−0.59 to 0.08]; *p* = 0.015; rho = −0.49, N = 20, 95% CI [−0.82 to 0.00]; *p* = 0.028, respectively), Δ TG (overall cohort rho = −0.344, N = 41, 95% CI [−0.55 to 0.12]; *p* = 0.028; MHO rho = −0.508, N = 20, 95% CI [−0.77 to −0.10]; *p* = 0.022), and Δ total cholesterol (MHO rho = −0.461, N = 20, 95% CI [−0.75 to −0.03]; *p* = 0.040). Δ MFN2-s1 (60 kDa) correlated negatively with Δ SBP (rho = −0.308, N = 43, *p* = 0.045) and positively with Δ hemoglobin in both the total (rho = 0.376, N = 40, *p* = 0.017) and MUO cohorts (rho = 0.483, N = 20, *p* = 0.031).

Additionally, Δ PINK1 mRNA was associated with Δ uric acid in the total group (rho = 0.362, N = 39, 95% CI [0.03 to 0.66]; *p* = 0.023), with Δ waist-to-hip ratio (rho = 0.460, N = 20, 95% CI [0.00 to 0.79]; *p* = 0.041) in MHO, and inversely with Δ glucose (rho = −0.590, N = 20, 95% CI [−0.87 to −0.15]; *p* = 0.006) in MHO, and with Δ creatinine (rho = −0.465, N = 19, 95% CI [−0.79 to −0.01]; *p* = 0.045) in MUO. In MHO, Δ FL-PINK1 protein was associated with Δ uric acid (rho = 0.586, N = 20, 95% CI [0.27 to 0.85]; *p* = 0.007), Δ ALT (rho = 0.446, N = 20, 95% CI [0.10 to 0.69]; *p* = 0.049), and Δ microalbuminuria (rho = 0.670, N = 17, 95% CI [0.33 to 0.84]; *p* = 0.003). The Δ mature PINK1 was inversely correlated with Δ weight (rho = −0.352, N = 43, 95% CI [−0.60 to −0.05]; *p* = 0.020) and Δ BMI (rho = −0.326, N = 43, 95% CI [−0.58 to −0.03]; *p* = 0.033) in the overall group.

Finally, Δ COX IV levels correlated with Δ BMI in MHO (rho = −0.503, N = 22, 95% CI [−0.78 to −0.08]; *p* = 0.017) and MUO (rho = 0.482, N = 21, 95% CI [−0.04 to −0.78]; *p* = 0.027). No significant associations were found with COX IV mRNA expression or for TFAM mRNA and protein levels.

## 4. Discussion

The main findings of this study were as follows: (i) MHO and MUO differ markedly in mitochondrial dynamics, with MUO showing a distinct pattern of MFN2 degradation and lower levels of other mitochondrial-dynamics-related proteins. (ii) Following a 12-month LSM program, MUO appear to benefit more clearly from the intervention, likely due to a larger margin for improvement. They saw increased OPA1-L, reduced MFN2 degradation products, and increased COX IV protein levels. In MHO, there was decreased Δ-PINK1, suggesting a reduced mitophagy demand. (iii) Changes in mitochondrial dynamics were associated with clinical improvements—most notably an increase in HDL in both groups—while anthropometric improvements occurred only in the MUO group.

Importantly, these findings provide clinically relevant evidence that mitochondrial adaptations to lifestyle modification are strongly influenced by metabolic phenotype in older adults with obesity. By directly comparing MHO and MUO participants, this study demonstrates that similar lifestyle interventions can induce distinct mitochondrial remodeling patterns, with implications for risk stratification and personalized lifestyle-based management in older populations.

Many studies in chronic and metabolic diseases use PBMCs to understand mitochondrial health, functional modulation, remodeling, and mitochondrial stimulation after lifestyle interventions [16,17]. This study, conducted in PBMCs, was designed primarily to evaluate phenotype-specific differences in mitochondrial fusion and mitophagy markers and their response to lifestyle modification. The results suggest that the baseline metabolic phenotype not only influences the magnitude but also the qualitative nature of mitochondrial remodeling in response to LSM, with greater structural recovery and clinically meaningful benefits observed in individuals with more pronounced initial impairments.

Consistent with prior LSM trials in different populations [18,19], anthropometric features remain largely stable after LSM in MHO, while MUO lost 3.5% of body weight, reduced their BMI by 2.4%, and saw smaller waist circumferences. These improvements were accompanied by a modest but significant increase in HDL, supporting a shift toward a less atherogenic profile with a potential effect on reducing the risk of heart disease.

Several studies have elucidated mechanisms linking mitochondrial dysfunction to obesity and aging [20,21]. Unlike most studies in adipocytes, PBMCs are a feasible and minimally invasive tissue that can provide systemic and longitudinal insights.

LSMs based on MedDiet and/or PA are known to modulate both mitochondrial health and activity in several tissues and diseases [22,23]. Mitochondrial dynamics encompass fission, fusion, biogenesis, mitophagy, and transport. This study focused on OPA1 and MFN2 fusion proteins, TFAM (biogenesis), and (PINK1) mitophagy [8]. Furthermore, COX IV levels were evaluated to analyze effects on the mitochondrial respiratory chain.

Despite the phenotypic instability of the MHO phenotype over time (transient state), no participants changed their baseline phenotype during the intervention. This is likely because the criteria used in this study were very strict and it would have proven quite difficult for a subject to transition from one phenotype to the other. Furthermore, the subjects were closely monitored by nutritionists and physical activity monitors, who helped MHO subjects maintain this phenotype. Nevertheless, the baseline mitochondrial health of these individuals may reflect a more plastic and intervention-responsive pre-pathological condition compared to the established dysfunction observed in the MUO phenotype.

OPA1, located in the inner mitochondrial membrane, is a master regulator of mitochondrial fusion, cristae stability, and OXPHOS, and participates in the maintenance of mtDNA and in apoptosis [24]. It is also essential for adipose tissue expansion, lipid biosynthesis, thermogenic regulation, and lipolysis [25,26]. Although there are several variants of OPA1, two main forms of 100 and 80 kDa (OPA1-L and OPA1-S) [27] are usually detected by Western blot. The results revealed that the 12-month LSM was associated with an improvement of mitochondrial fusion in both populations. OPA1-L tends to increase in both MUO and MHO, in whom the change is more noteworthy, possibly since they showed significantly less OPA1 at baseline. OPA1-S, involved in mitochondrial fusion as well in energetics [28], also increased in both groups in a non-significant manner.

MFN2, essential for mitochondrial structure and signaling [29], was also reduced in MUO’s PBMCs compared to MHO, in line with prior findings that MFN2 downregulation accompanies obesity and metabolic dysfunction [30]. Moreover, MUO showed two additional bands, which likely represent putative MFN2 degradation products. After the LSM, these bands decreased, suggesting reduced MFN2 degradation and partial recovery of fusion competence. However, alternative explanations such as splice variants or antibody cross-reactivity cannot be excluded

Interestingly, protein and mRNA levels often diverge. This mismatch may reflect compensatory transcriptional responses whereby increased mRNA expression does not necessarily translate into higher protein levels due to abnormalities in the efficiency of post-transcriptional and translational regulatory mechanisms. In addition, enhanced protein turnover or degradation of mitochondrial proteins may further contribute to this dissociation. This interpretation is supported by previous studies that report findings similar to this work’s, namely discordant gene–protein responses under conditions of metabolic stress, exercise, and aging in muscle [31] or adipose tissues [32].

The PINK1–Parkin pathway is mainly involved in mitophagy, among other functions. PINK1 undergoes proteolytic processing to yield the FL-PINK1 and cytosolic isoforms (mature PINK1 and PINK1-s), which bind to PARKIN and inhibit mitophagy [33]. These forms are normally transient and barely detectable in healthy PBMCs, whereas altered ratios have been linked to metabolic and age-related dysfunctions [34,35].

In this cohort, MHO predominantly showed mature PINK1 and PINK1-s with absent FL-PINK1, consistent with efficient processing, whereas MUO displayed persistent FL-PINK1 with additional degradation products, suggesting impaired mitophagy. After LSM, MHO saw reduced mature PINK1 and its mRNA, reflecting lower mitophagy demand, while MUO showed non-significant changes, indicating resistance of mitophagy dysfunction to intervention despite systemic clinical improvements in this phenotype. Altogether, these results suggest that LSM is associated with the preservation of mitophagy efficiency in healthier individuals while only partially attenuating mitophagy-related stress markers in MUO.

Regarding biogenesis, TFAM methylation in PBMCs has been associated with insulin resistance [36] and caloric restriction increases its expression in skeletal muscle in individuals with overweight—but not with obesity [37]. In this study, TFAM remained unchanged in PBMCs in both groups. These findings align with previous evidence that TFAM is closely regulated and may not respond robustly to moderate lifestyle changes, especially in non-muscular tissues such as blood cells [38].

The electron transport chain plays a central role in cellular energy production and homeostasis. Its dysfunction can contribute to the metabolic alterations and its activity is known to decline by 10% in each decade of age [39]. Although no significant differences in COX IV protein levels between MHO and MUO were found in this study, COX IV mRNA expression was significantly higher in MUO at baseline, potentially reflecting a compensatory transcriptional response to mitochondrial dysfunction. After the LSM, COX IV protein levels significantly increased in MUO participants, while COX IV mRNA expression decreased. This inverse regulation may reflect enhanced translation efficiency, post-transcriptional regulation, or stabilization of respiratory complexes in response to improved mitochondrial function, as is reported in other exercise-based or dietary interventions [40]. In MHO, no changes were found in mitochondrial respiratory activity, likely for the same reasons expressed above for the absence of changes in TFAM.

Correlation analyses were performed as exploratory secondary analyses to generate hypotheses regarding potential links between mitochondrial adaptations and clinical or lifestyle-related changes. Lifestyle interventions are known to influence mitochondrial function and dynamics, leading to systemic metabolic benefits [41]. Across the entire cohort, it was observed that changes in OPA1 mRNA were negatively associated with BMI reduction. In MHO, decreases in total OPA1 protein also correlated with reductions in BMI and ALT. Similar associations between improved fusion capacity and reduced adiposity have been reported in both humans and preclinical models [25,42].

MFN2 variations correlated inversely with triglycerides and cholesterol, while in MUO they also correlated inversely with DBP and hemoglobin, supporting its role in lipid and vascular regulation. For PINK1, several protein forms and its mRNA were associated with clinical outcomes, including positive correlations with uric acid and ALT and negative correlations with BMI, weight, glucose, alkaline phosphatase, creatinine, and waist-to-hip ratio, depending on the phenotype. These associations indicate that modulation of mitophagy may influence metabolic, hepatic, and renal parameters, as is observed in metabolic disorders, where impaired PINK1 signaling contributes to mitochondrial dysfunction and worsened metabolic outcomes [43].

Previous studies have demonstrated that physical activity can restore mitochondrial quality control mechanisms, enhancing fusion and mitophagy pathways, particularly in metabolically compromised individuals [44,45]. The increases in physical activity (RAPA score) observed in this study were linked to higher OPA1-L and MFN2 (80 kDa) levels and to changes in PINK1 forms in the overall cohort, reinforcing exercise as a key driver of mitochondrial remodeling

While PBMCs offer a practical and minimally invasive model for assessing mitochondrial bioenergetics, their functional profile does not strictly mirror the dynamics of highly metabolically active tissues, such as skeletal muscle or adipose tissue. Evidence indicates that mitochondrial respiration in PBMCs may not directly correlate with muscle-specific adaptations, suggesting that these cells are not direct surrogates for local tissue metabolism [46]. Nevertheless, PBMC bioenergetics have been shown to be associated with systemic phenotypes, including physical performance and inflammatory status, which implies that they can serve as a proxy for global or immune-cell mitochondrial health rather than local metabolic shifts [47]. Consequently, while this study’s findings provide valuable insights into systemic adaptations to LSM, these results should be interpreted as markers of circulating or immune-related mitochondrial health rather than direct indicators of muscle or adipose tissue dynamics.

Finally, although this 12-month observational cohort study provides robust temporal data, the lack of a non-intervention control group means that definitive causal conclusions cannot be drawn. Consequently, the results should be interpreted as associations between LSM and mitochondrial outcomes and findings should be interpreted with caution.

This study suggests that a “one-size-fits-all” approach to the study of obesity in older adults is outdated. First, the mitochondria used as biomarkers demonstrate that mitochondrial health in PBMCs can serve as a systemic “bioenergetic sensor.” These biomarkers allow observation of whether a lifestyle intervention is having an effect at a cellular level, even before significant weight loss is visible. This study also evaluates the importance of risk stratification in aging, since older adults are a vulnerable population. Understanding their metabolic phenotype along with lifestyle changes would allow for treating multi-organ obesity and managing the response to diet and exercise before detrimental effects emerge in older adults. Additionally, “mitoprotective” therapies can be developed by identifying specific mitochondrial impairments associated with these MHO/MUO phenotypes, which may allow exploration of the potential reversal of the diseased state to a healthy state.

## 5. Conclusions

This study’s findings indicate that weight loss achieved through healthy LSM enhances mitochondrial function in both MHO and MUO older adults, potentially mitigating obesity-related health risks in these vulnerable cohorts. This improvement helps reduce the health risks of obesity in older adults. However, these data show that specific mitochondrial deficits inherent to each metabolic phenotype influence how a patient responds to lifestyle changes. These findings suggest that clinical interventions should be tailored to an individual’s metabolic phenotype to maximize the benefits of lifestyle modifications in older adults.

Mitochondrial changes in PBMCs are highly correlated with systemic health. However, as this study is observational and uses a LSM without a direct molecular “knock-in/knock-out” model, it cannot be definitively stated that correlations between mitochondrial improvements and weight loss suggest a link. They do not prove that the weight loss caused the mitochondrial improvement, since it could be due to the increased exercise or dietary changes. However, this work serves as a pioneering study that sets the stage for future mechanistic trials, like those using pharmacological mitochondrial boosters.

## 6. Study Limitations

This work’s relatively small sample size (n = 43) may limit its statistical power and missing data in some participants restricted complete-case analyses. The exploratory approach involving multiple correlation analyses increases the risk of type I error, and the observational study design precludes causal inference. When discussing null findings, particularly in the MHO group, it is essential to address whether the lack of statistical significance is due to true biological stability or a lack of statistical power. The high degree of inter-individual variability often means that subtle improvements in healthy populations require larger cohorts to detect. This work is a longitudinal mitochondrial research study that does have this limitation of sample size. Finally, as the study population consisted exclusively of older adults with obesity, the generalizability of the findings to other age groups or metabolic conditions may be limited.

## Figures and Tables

**Table 1:** Anthropometric and analytical parameters of MHO, MUO, and the total population at baseline and after the 12-month LSM intervention.

	MHO (N = 22)					MUO (N = 21)			Overall (N = 43)			
	Baseline	After LSM	Δ	Cohen’s d	Baseline	After LSM	Δ	Cohen’s d	Baseline	After LSM	Δ	Cohen’s d
**Age (years)**	72 ± 5				69 ± 3				70 ± 4			
**Sex (Female)**	15 (68.2%)				15 (71.4%)				30 (69.8%)			
**Weight (kg)**	83.0 ± 11.5	83.3 ± 12.1	0.32	0.13	86.2 ± 11.1	83.4 ± 10.2	−2.79	−0.88 *	84.6 ± 11.2	83.4 ± 11.1	−1.20	−0.37 *
**BMI (kg/m^2^)**	33.7 ± 2.5	33.9 ± 2.9	0.18	0.18	33.8 ± 3.7	33.0 ± 3.6	−0.82	−0.70 *	33.7 ± 3.2	33.4 ± 3.3	−0.31	−0.26
**Waist (cm)**	104.4 ± 7.7	105.3 ± 9.1	0.88	0.10	115.3 ± 11.0	110.9 ± 9.5	−4.38	−0.80 *	109.7 ± 10.8	108.1 ± 9.6	−1.62	−0.34 *
**Hip (cm)**	114.1 ± 8.3	115.0 ± 9.7	0.91	0.11	114.4 ± 8.3	113.1 ± 8.9	−1.29	−0.28	114.2 ± 8.2	114.0 ± 9.2	−0.19	−0.07
**Waist-to hip**	0.919 ± 0.1	0.918 ± 0.1	0.00	−0.00	1.01 ± 0.07	0.98 ± 0.06	−0.03	−0.61 *	0.96 ± 0.09	0.95 ± 0.09	−0.01	−0.31
**Glc (mg/dL)**	86.7 ± 12.3	86.1 ± 12.02	−0.63	−0.17	118.1 ± 27.6	120.9 ± 34.1	2.76	0.07	101.6 ± 26.1	103.9 ± 30.5	2.24	−0.01
**TC (mg/dL)**	208.5 ± 24.6	212.6 ± 30.6	4.50	0.06	200.6 ± 42.0	198.9 ± 48.8	−1.67	−0.05	204.4 ± 34.0	205.6 ± 41.0	1.17	−0.00
**HDL (mg/dL)**	59.7 ± 15.3	71.5 ± 31.4	11.77	0.40 *	52.6 ± 13.1	54.7 ± 12.0	2.14	0.56 *	56.2 ± 14.5	62.9 ± 24.7	6.67	0.33 *
**LDL (mg/dL)**	127.3 ± 21.2	126.6 ± 37.7	−0.72	−0.10	118.9 ± 38.1	116.7 ± 41.5	−2.29	−0.07	123.2 ± 30.6	121.5 ± 35.9	−1.72	−0.08
**TG (mg/dL)**	103.3 ± 57.3	103.8 ± 37.7	0.53	−0.08	145.3 ± 43.0	137.1 ± 37.2	−8.19	−0.22	123.8 ± 54.5	120.9 ± 40.6	−2.94	−0.15
**HbAc1 (%)**	5.70 ± 0.3	5.73 ± 0.3	0.03	0.12	6.1 ± 0.7	5.9 ± 0.7	−0.21	−0.40	5.9 ± 0.6	5.8 ± 0.6	−0.08	−0.24

Values are expressed as means ± SD or N (%).(*, *p* < 0.05).Abbreviations: Metabolically Healthy Obese older adults: MHO; Metabolically Unhealthy Obese older adults: MUO; BMI: Body Mass Index; Glc: Glucose; TC: Total Cholesterol; HDL: High-Density Lipoprotein; LDL: Low-Density Lipoprotein; TG: Triglycerides; HbAc1: glycated hemoglobin.

**Table 2:** MedDiet adherence and PA scores at baseline and 12 months in MHO and MUO participants.

	MHO (N = 22)				MUO (N = 21)				Overall (N = 43)			
	Baseline	After LSM	Δ	Cohen’s d	Baseline	After LSM	Δ	Cohen’s d	Baseline	After LSM	Δ	Cohen’s d
**MEDAS Index**												
**Mean ± SD points**	8.82 ± 2.26	9.77 ± 1.80	0.95	0.512 *	8.14 ± 1.74	10.29 ± 1.23	2.14	−1.30 *	8.82 ± 2.26	9.77 ± 1.80	0.95	0.512 *
**Low Adherence (n (%))**	2 (9.1)	1 (4.8)			2 (9.5)	0 (0)			4 (9.3)	1 (2.4)		
**Moderate Adherence (n (%))**	10 (45.5)	7 (33.3)			16 (76.2)	7 (33.3)			26 (60.5)	14 (33.3)		
**High Adherence** **(n (%))**	10 (45.5)	13 (61.9)			3 (14.3)	14 (66.7)			13 (30.2)	27 (64.3)		
**RAPA score**												
**Mean ± SD points**	4.82 ± 1.71	2.95 ± 1.59	−1.86	−0.888 *	1.62 ± 0.80	2.10 ± 0.89	0.48	0.546 *	3.26 ± 2.09	2.53 ± 1.35	−0.72	−0.362 *
**Sedentary (n (%))**	1 (4.5)	6 (27.3)			12 (57.1)	7 (33.3)			13 (30.2)	13 (30.2)		
**Underactive (n (%))**	0 (0)	0 (0)			5 (23.8)	5 (23.8)			5 (11.6)	5 (11.6)		
**Underactive regular (light activities) (n (%))**	5 (22.7)	11 (50)			4 (19)	9 (42.9)			20 (46.5)	20 (46.5)		
**Underactive regular**	6 (27.3)	2 (9.1)			0 (0)	0 (0)			2 (4.7)	2 (4.7)		
**Active (n (%))**	10 (45.5)	3 (13.6)			0 (0)	0 (0)			3 (7)	3 (7)		

Mediterranean Diet (MedDiet) adherence was assessed using the MEDAS (classifies participants based on their level of adherence to the MedDiet as low, moderate, or high, according to the scores obtained in the questionnaire) and physical activity using the RAPA questionnaire (classifies participants based on their physical activity level as Underactive, Underactive Regular (light activities), or Active, according to the scores obtained in the questionnaire). Values (points) are expressed as mean ± SD and *p*-values (*, *p* < 0.05), or number of patients (n) and percentage (%) of them in the different categories, refer to intra-group comparisons between baseline and 12-month follow-up. Effect sizes were calculated using Cohen’s d.

## Data Availability

The original contributions presented in this study are included in the article/Supplementary Materials. Further inquiries can be directed to the corresponding author.

## Supplementary Materials

The following supporting information can be downloaded at: https://www.mdpi.com/article/10.3390/nu18030475/s1, Table S1. List of primary and secondary antibodies used in this work, Table S2. List and features of primers used in this study, Figure S1: (A) Representative Western blot and relative quantification of TFAM and (B) RT-qPCR analysis of TFAM mRNA relative expression in MHO and MUHO PBMCs at baseline and after 12-month LSM intervention. Data are expressed as mean ± SEM.

## Abbreviations

The following abbreviations are used in this manuscript:

ALT	Alanine Transaminase
AP	Alkaline Phosphatase
BMI	Body Mass Index
BSA	Bovine Serum Albumin
COX IV	Cytochrome c Oxidase Subunit IV
DBP	Diastolic Blood Pressure
DNA	Deoxyribonucleic Acid
EDTA	Ethylenediaminetetraacetic Acid
FBS	Fetal Bovine Serum
GAPDH	Glyceraldehyde-3-Phosphate Dehydrogenase
GGT	Gamma-Glutamyl Transferase
Hb	Hemoglobin
HbAc1	Glycated Hemoglobin
HDL	High-Density Lipoprotein
HR	Heart Rate
LDL	Low-Density Lipoprotein
LSM	Lifestyle Modification
MedDiet	Mediterranean Diet
mRNA	Messenger RNA
mtDNA	Mitochondrial DNA
MFN2	Mitofusin 2
MHO	Metabolically Healthy Obesity
MUFA	Monounsaturated Fatty Acids
MUO	Metabolically Unhealthy Obesity
OPA1	Optic Atrophy Type 1
PA	Physical Activity
PBMCs	Peripheral Blood Mononuclear Cells
PBS	Phosphate-Buffered Saline
PINK1	PTEN-Induced Kinase 1
PUFA	Polyunsaturated Fatty Acids
RAPA	Rapid Assessment of Physical Activity
RT-qPCR	Real-Time Quantitative Polymerase Chain Reaction
SBP	Systolic Blood Pressure
SEM	Standard Error of the Mean
SFA	Saturated Fatty Acids
TFAM	Mitochondrial Transcription Factor A
TG	Triglycerides
T2DM	Type 2 Diabetes Mellitus
UA	Uric Acid
Vit D	Vitamin D
WB	Western Blot
WHO	World Health Organization
