# Supplementary material for: Phenotype-Specific Mitochondrial Responses to Mediterranean Diet and Exercise in Elderly Obesity
Source: Nutrients. 2026 Feb 1;18(3):475. doi: 10.3390/nu18030475 (PMC12899043; doi:10.3390/nu18030475)
Supplement: Supplementary file 1 [file nutrients-18-00475-s001.zip › suplementary/TableS1.pdf]

	OPA1 (1E81D9)	MFN2 (4H8)	PINK1 (D8G3)	TFAM (D5C8)	COX IV (3E11)	B-ACTINA (C4)	ANTI-MOUSE	ANTI-RABBIT
**Manufacturer**	ABCAM	ABNOVA	CELL SIGNALING	CELL SIGNALING	CELL SIGNALING	SANTA CRUZ	SIGMA ALDRICH	SIGMA ALDRICH
**Reference**	AB119685	H00009927-M03	6946	8076	4850	SC-47778	GENA931	A0545
**MW (kDa)**	100-80	86, 50	63, 55, 42	24	17	42	-	-
**Dilution**	1:1000	1:1000	1:1000	1:1000	1:1000	1:1000	1:5000	1:5000
**Host**	Mouse	Mouse	Rabbit	Rabbit	Rabbit	Mouse	Sheep	Goat
**Resolving Gel**	10,00 %	10,00 %	10,00 %	15,00 %	15,00 %	-	-	-
**Storage**	4 ºC	-20 ºC	-20 ºC	-20 ºC	-20 ºC	4 ºC	4 ºC	-20 ºC
