# Supplementary material for: Phenotype-Specific Mitochondrial Responses to Mediterranean Diet and Exercise in Elderly Obesity
Source: Nutrients. 2026 Feb 1;18(3):475. doi: 10.3390/nu18030475 (PMC12899043; doi:10.3390/nu18030475)
Supplement: Supplementary file 1 [file nutrients-18-00475-s001.zip › suplementary/TableS2.pdf]

Gene	Sequence(5'-3')		Tm (ºC)
**GAPDH**	**Forward**	TCGGAGTCAACGGATTTG	62.4
	**Reverse**	CAACAATATCCACTTTACCAGAG	59.1
**MFN2**	**Forward**	CTTCTTTGTGTCTGCTAAGG	56.7
	**Reverse**	CTGAAACTCAAACATCCTCAC	58.8
**OPA1**	**Forward**	TGAGTTGGAATGCAATGATG	62.2
	**Reverse**	GTTGCCTTAAAGTATTTGCG	58.9
**TFAM**	**Forward**	GAAAGATTCCAAGAAGCTAAGG	59.9
	**Reverse**	CGTCCAACTTCAATCATTTG	60.6
**PINK1**	**Forward**	CGTTATGAAGAACTATCCCTG	57.8
	**Reverse**	CAAGGATGTTGTCGGATTTC	61.9
**COX IV**	**Forward**	ATTGAAGGAGAAGGAGAAGG	58.9
	**Reverse**	CTCCTTGAACTTAATGCGATAC	59.3
