# Supplementary figures and images for: Phenotype-Specific Mitochondrial Responses to Mediterranean Diet and Exercise in Elderly Obesity
Source: Nutrients. 2026 Feb 1;18(3):475. doi: 10.3390/nu18030475 (PMC12899043; doi:10.3390/nu18030475)

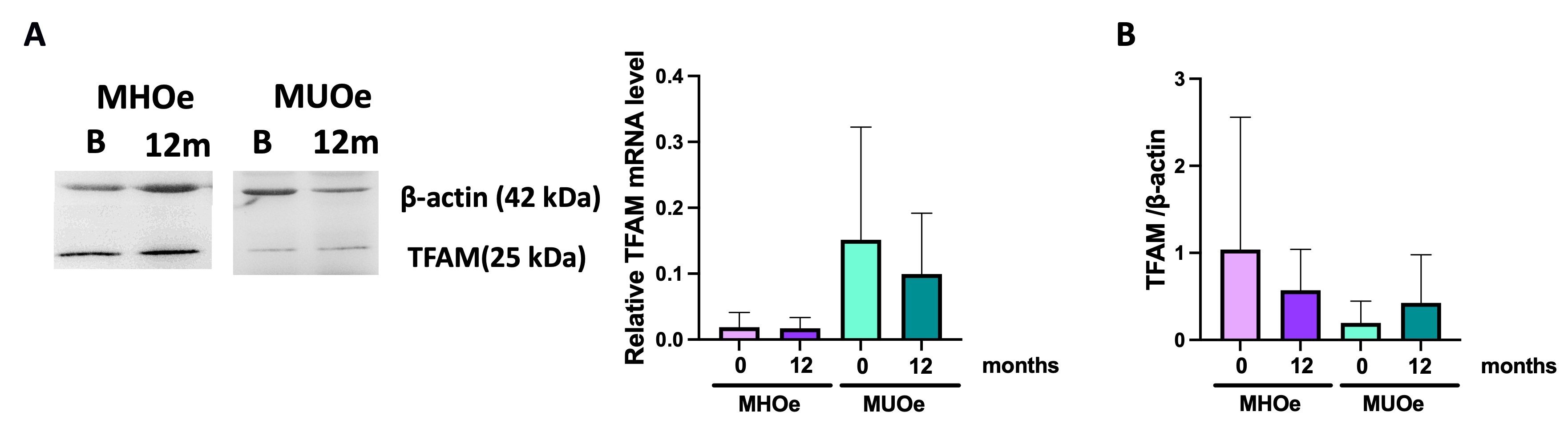

Supplement: Supplementary file 1 [file nutrients-18-00475-s001.zip › suplementary/Figure S1.png]
